# Aluminum Nitride to Silicon Direct Bonding for an Alternative Silicon-On-Insulator Platform

**DOI:** 10.1021/acsami.1c09535

**Published:** 2021-08-04

**Authors:** Jani Kaaos, Glenn Ross, Mervi Paulasto-Kröckel

**Affiliations:** Department of Electrical Engineering and Automation, Aalto University, P.O. Box 13500, FIN-00076 Aalto, Finland

**Keywords:** silicon-on-insulator, direct bonding, aluminum nitride, microelectromechanical systems, plasma activation, surface chemistry, surface topography

## Abstract

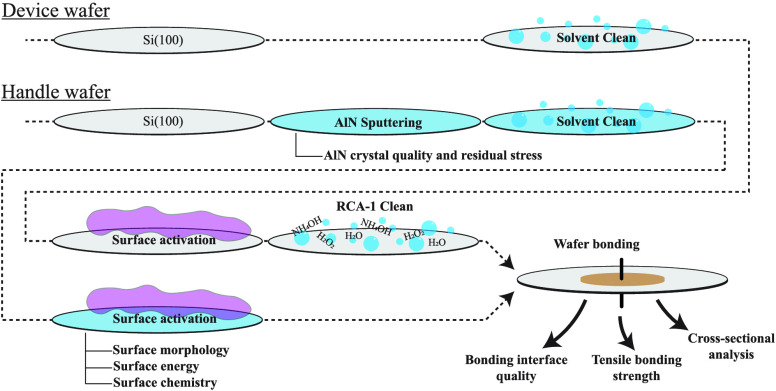

The next generation of microelectromechanical systems (MEMS) requires new materials and platforms that can exploit the intrinsic properties of advanced materials and structures, such as materials with high thermal conductivity, broad optical transmission spectra, piezoelectric properties, and miniaturization potential. Therefore, we need to look beyond standard SiO_2_-based silicon-on-insulator (SOI) structures to realize ubiquitous MEMS. This work proposes using AlN as an alternative SOI structure due to several inherent material property advantages as well as functional advantages. This work presents the results of reactively sputtered AlN films on a Si handle wafer bonded with a mirror-polished Si device wafer. Wafer bonding was achieved by using hydrophilic wafer bonding processes, which was realized by appropriate polymerization of the prebonding surfaces. Plasma activation of the AlN surface included O_2_, Ar, SF_6_, SF_6_ + Ar, and/or SF_6_ + O_2_, which resulted in a change in the chemical and topography state of the surface. Changes in the AlN surface properties included enhanced hydrophilicity, reduced surface roughness, and low nanotopography, components essential for successful hydrophilic direct wafer bonding. Wafer bonding experiments were carried out using promising surface activation methods. The results showed a multilayered bonding interface of Si(Device)/SiO_2_/ALON/AlN/Si(Handle) with fluorine in the aluminum oxynitride layer from the proceeding AlN surface activation process. More notably, this work provided wafer bonding tensile strength results of the AlN alternative SOI structure that compares with the traditional SiO_2_ SOI counterpart, making AlN to Si direct bonding an attractive alternative SOI platform.

## Introduction

The silicon-on-insulator (SOI) platform based on silicon dioxide has been an enabler of CMOS-based technologies through to silicon-based microelectromechanical systems (MEMS). In the transistor domain, SOI has helped realize partially and fully depleted CMOS transistor technology, giving superior transistor electrostatic control and reduced parasitic capacitances. In silicon MEMS technologies, SOI has made possible devices that are able to sense, control and actuate by integrated cavities and superior etch stop capabilities reducing fabrication complexity. However, as MEMS technologies move beyond the consumer-driven boom of the last decade, new materials and platforms are required to realize the next generation of ubiquitous MEMS.^1−5^

New materials and platforms are required to achieve technological developments in all subfields of MEMS, such as micro-opto-electromechanical systems (MOEMS), radio-frequency-MEMS(RF-MEMS), and BioMEMS. New materials allow application designers to exploit their intrinsic material properties, such as their thermomechanical, optical, and electrical properties, to name a few. A specific example of this, and one of the motivations for this work, is the inherent limitations of SiO_2_. SiO_2_ suffers from poor thermal conductivity. This becomes a significant disadvantage as thermal designs are becoming increasingly challenging due to miniaturization, density, and the increasing number of thermal interfaces.

Another possible SOI configuration is to use an alternative insulation material. One potential material is AlN, which has several unique advantages over its SiO_2_ counterpart.^6^ AlN is a crystalline material, as opposed to amorphous SiO_2_ used in SOI, that has highly tailorable thermomechanical properties depending on the deposition technique and conditions. Reactive sputtering is one of the most common methods for depositing high-crystal quality AlN and is a commonly used CMOS compatible process.^7,8^ This process allows for tailoring of the AlN film properties, such as morphology, crystal quality, and residual stresses. These properties undoubtedly affect the functionality, processability, and thermomechanical reliability of an alternative SOI platform.

A favorable property of a dielectric layer in an SOI structure is the thermal conductivity and its heat spreading capability. AlN has a significantly higher thermal conductivity (319 W/m·K) compared with traditional thermal SiO_2_ (1.4 W/m·K).^9,10^ In addition to the thermal properties, there are also considerable mechanical differences between AlN and SiO_2_ where the hardness of AlN is higher than that of SiO_2_ and the fracture strength of AlN is almost twice that of SiO_2_, 1.54 and 0.81 GPa, respectively.^11,12^ All of these are advantageous for the structural integrity of an SOI platform. Another noteworthy attribute of AlN is that it is a piezoelectric material that is highly established in the field of piezo-based MEMS, which could potentially lead to embedded piezo-MEMS, in an alternative SOI configuration. In the field of silicon photonics and MOEMS, AlN has a broad transmission spectrum, ranging from the near-infrared to ultraviolet. Additionally, AlN has a relatively strong electro-optic coefficient, making it advantageous for efficient low power electro-optic modulation. All of these functional properties open a broad range of development possibilities when built with a high-crystal quality, low-impurity AlN-based SOI platform.^13−15^

SOI wafers are achieved by various processes, one being direct wafer bonding with an etch/polish back process of the device silicon layer. The advantage of this approach is that the device wafer retains the crystalline quality of the prebonded wafers, with the ingot cutting process determining the device wafer crystal orientation. Direct bonding is the process of joining two heterogeneous or homogeneous materials, which have low flatness and roughness. A subset of direct bonding is hydrophilic wafer bonding, which involves the polymerization of the prebonded mating surfaces, enabling contact forces that drive the bonding process. Direct hydrophilic wafer bonding has the advantages of being a low temperature process that allows initial intersurface polymerization to occur via the presurface chemistry, followed by diffusion-based mass transport at elevated temperatures. To fully understand how the bonding process proceeds, there are two critical components affecting the bonding performance: (i) the surface chemistry and (ii) surface topography of the prebonding surfaces.^16,17^

Early rudimentary studies into sputtered AlN–Si direct bonding performed by Bengtsson *et al.*(18,19) revealed that bonding of these two materials was indeed possible, although resulted in relatively low bonding strengths. The bonds required a high postannealing temperature to reinforce the strength, negating the advantages of the low-temperature hydrophilic process. In these studies, no prebonding plasma treatments were undertaken. Men *et al.*(20,21) examined the possibility of using electron beam-evaporated AlN bonded to Si using hydrophilic wafer bonding. Their results showed that bonding was successful; however, no measure of bonding strength was presented. The structure of AlN was amorphous that would undoubtedly affect the thermomechanical performance of the film. Additionally, a high-temperature annealing step, as high as 1100 °C, was used to achieve the ion-cutting process for thinning the device wafer. No plasma activation of the surface was made prebond, most likely due to the very low surface roughness of their amorphous film. Recently, Olver^22^ studied AlN–AlN direct bonding as an adhesion layer to fabricate sapphire quantum wells. The author sputter-deposited thin AlN films on wafers and undertook a multistep plasma activation utilizing combinations of O_2_, Ar, and SF_6_ and DIW rinse followed by wafer bonding. AlN–AlN bonds were successful, and the authors concluded that the combination of both surface topography and activation is critical in obtaining a successful bond. Bao *et al.*(23) reported an AlN–AlN direct bond as a means to fabricate an SOI substrate with enhanced thermal conductivity. The thin AlN films were deposited using atomic layer deposition that was followed by an outgassing at elevated temperatures, activated via argon, and bonded at low temperature. Enhanced heat conductivity with respect to SiO_2_ and Al_2_O_3_ was achieved, albeit the results were abated due to a relatively thick interfacial bonding layer. The plasma activation appeared to have induced a hydrophilic alumina surface layer on AlN that was capable of the intersurface polymerization.

The knowledge gap in heterogeneous AlN to Si hydrophilic direct wafer bonding is a combination of both plasma-activated AlN surface characterization, including the surface topography and chemistry, and how these parameters interact during bonding to a mirror-polished and RCA-1 cleaned Si wafer. This work demonstrates the use of AlN as an alternative SOI structure that exhibits comparable tensile strength properties to its SiO_2_ counterpart. A range of surface activation processes and their impact on the surface chemistry, topography, and bonding performance were studied, to shed light on the underlying chemical and mechanical bonding mechanisms. Finally, a cross-sectional analysis presents the microstructure of the bonding interface of a sample that resulted in the highest tensile strength. The results in this work help to further our understanding of surface activation methods and their impact, which will enable high-strength hydrophilic wafer-level bonding.

## Materials and Methods

Ten aluminum nitride films were reactive sputter deposited onto 320 μm thick Si(100) wafers using a Von Ardenne CS 730 S sputtering system. AlN thin film target thicknesses ranged from 300 to 1200 nm. The stress state of the AlN film was measured using a WITec alpha300 RA+ Raman microspectrometer from the  phonon mode. The crystallinity of the films was characterized by X-ray diffraction (XRD) using a Rigaku SmartLab X-ray diffractometer. The surface roughness was characterized with an atomic force microscope (AFM) Bruker Dimension Icon. The uniformity of the films was characterized using a Semilab SE-2000 spectroscopic ellipsometer, and the chemical surface state was analyzed with both a Biolin Scientifics contact angle meter THETA and a Kratos Axis Ultra X-ray photoelectron spectrometer (XPS).

To study the impact of different surface activation methods, two wafers with a thicker AlN film were diced and activated utilizing an Oxford Instruments Plasmalab 80Plus RIE. The effect of the activation was characterized by using contact angle measurements, AFM scans, and XPS analysis.

**Figure 1:**
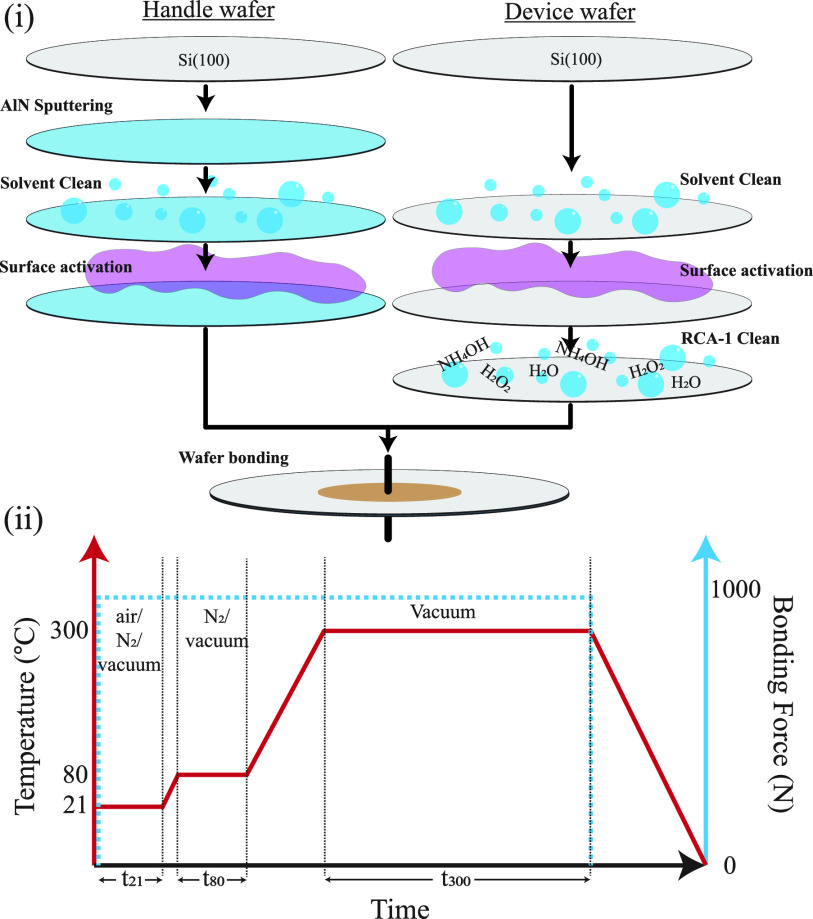
(i) Wafer bonding process flow, including AlN sputtering, cleaning, surface activation, and bonding steps and (ii) temperature–bonding force–time plot of the wafer bonding experiments.

To fabricate the alternative SOI platform, eight handle wafers with the deposited AlN films were activated and bonded onto the Si(100)-oriented surfaces of the Si device wafers. The activation and cleaning steps included a solvent cleaning protocol in an ultrasonic bath with subsequent spin drying, RIE activation on both faces, RCA-1 cleaning of the Si, and another round of spin drying. The bonding was initiated directly after the last cleaning step either using an AML-AWB wafer bonder or on a table under ambient conditions. Figure 1 shows the wafer bonding process flow, including the cleaning and surface activation steps.

The handle and device wafers were loaded into the wafer bonder and their flats were aligned, after which the bonding chamber was filled with nitrogen, air, or vacuum. The contact was initiated based on images from the bonder *in situ* infrared (IR) cameras, with a contact force of roughly 1 kN being applied to the wafers. Initially, the bonding process would develop at room temperature (RT) within the chamber for the duration *t*_21_, then, the temperature was raised to 80 °C for the duration *t*_80_, and finally, the temperature was raised to 300 °C for the duration *t*_300_. Next, the bonding force was released, heaters deactivated, and the chamber was passively left to slowly cool to RT. Four wafer-level bonding experiments were undertaken: a nonactivated bond made in the wafer bonder with progressive annealing stages (NA), a room temperature bond made in ambient air outside of the wafer bonder (RT), a vacuum bond made in the wafer bonder with progressive annealing stages (V), and a combined bond made in the activation process in ambient air but carried out in the wafer bonder with progressive annealing stages (C). In addition, postbonding annealing was performed on selected dies in a vacuum furnace at 600 °C, after which the tensile tests were repeated on the annealed samples (+A).

The bonded wafers were characterized by a Sonix HS3000 scanning acoustic microscope (SAM). The tensile strength was characterized by dicing the wafers to 25 mm^2^-sized dies, gluing them onto brass studs, and tensile tested in an MTS 858 Table System. The number of tensile test samples depended on the (i) yield of the wafer bonding process and (ii) success of the die mounting and gluing process. This included 14 dies for the NA, 5 dies for the RT, 8 dies for RT+A, 18 dies for the V, 27 dies for the V+A, 15 dies for the C, and 7 dies for the C+A. A more detailed description of the tensile test procedure can be found in the study by Ross *et al.*(6)

A combined wafer bond (C) diced die was prepared for high-magnification cross-sectional analysis. First, the diced chip was molded and cross-sectioned using a standard scanning electron microscope (SEM) and a metallographic grinding and polishing sample preparation method. The TEM lamella process was carried out using a dual-beam (FIB-SEM) JEOL JIB-4700F using an *in situ* lift-out process from the molded cross-section. Transmission electron microscopy (TEM), scanning transmission electron microscopy (STEM), and select area electron diffraction (SAED) were conducted using a JEOL JEM-2200FS Cs-corrected microscope, and energy-dispersive X-ray spectroscopy (EDS) was conducted using the JEOL JEM-2800, both operating at 200 kV.

## Results

The crystallographic properties of the as-deposited sputtered AlN were observed in both crystallinity 2θ = 36.1° ± 0.03° and *c*-axis out-of-plane growth orientation rocking curve FWHM(ω) = 2.3° ± 0.6°. Moreover, their bi-axial tensile residual stress was in the magnitude of 700 ± 300 MPa. To conclude, the characterization of the as-deposited AlN films, a convex shape of the films was observed; moreover, their uniformity was measured at 7 ± 1%.

On the AlN surface, large-area AFM scans of 400 μm^2^ were conducted and the surface waviness and roughness were deconvoluted. The obtained surface waviness components were *W*_q_ = 4 ± 2 Å and *W*_λ_ = 2 ± 1 μm, which should be elastically accommodated during the bonding process, and the surface roughness was *R*_q_ = 6–12 Å and *R*_λ_ = 230 ± 40 nm that requires mass transport to achieve an interfacial bonding contact. In addition, both water and hexadecane contact angles of the as-deposited AlN films (θ_w,AlN_ = 40°–90°; θ_h,AlN_ = 8°–36°) from each deposition batch, as well as a pristine silicon wafer, were recorded. A summary of the results can be seen in Table 1. The roughest films could be smoothened to 7 Å at best, via SF_6_-based RIE activation, that could be coupled with either argon or oxygen ion bombardment to induce hydrophilicity and remove surface contamination.

**Table 1:** Effect of AlN and Si Surface Activation on Both Surface Wetting and Roughness^a^

	θ_w_ (°)	θ_h_ (°)	*R*_q_ (Å)
AlN surface			
pristine AlN	57	30	12
O_2_	16	22	7
Ar	7	5	14
SF_6_	16	7	7
SF_6_ + O_2_	<5	41	9
SF_6_ + Ar	<5	26	7
Si surface			
pristine Si	29	9	2
O_2_ + RCA-1	<5	30	3

a Single point measurements were conducted on a diced wafer. The AFM scans are conducted over a 400 μm^2^ area.

**Figure 2:**
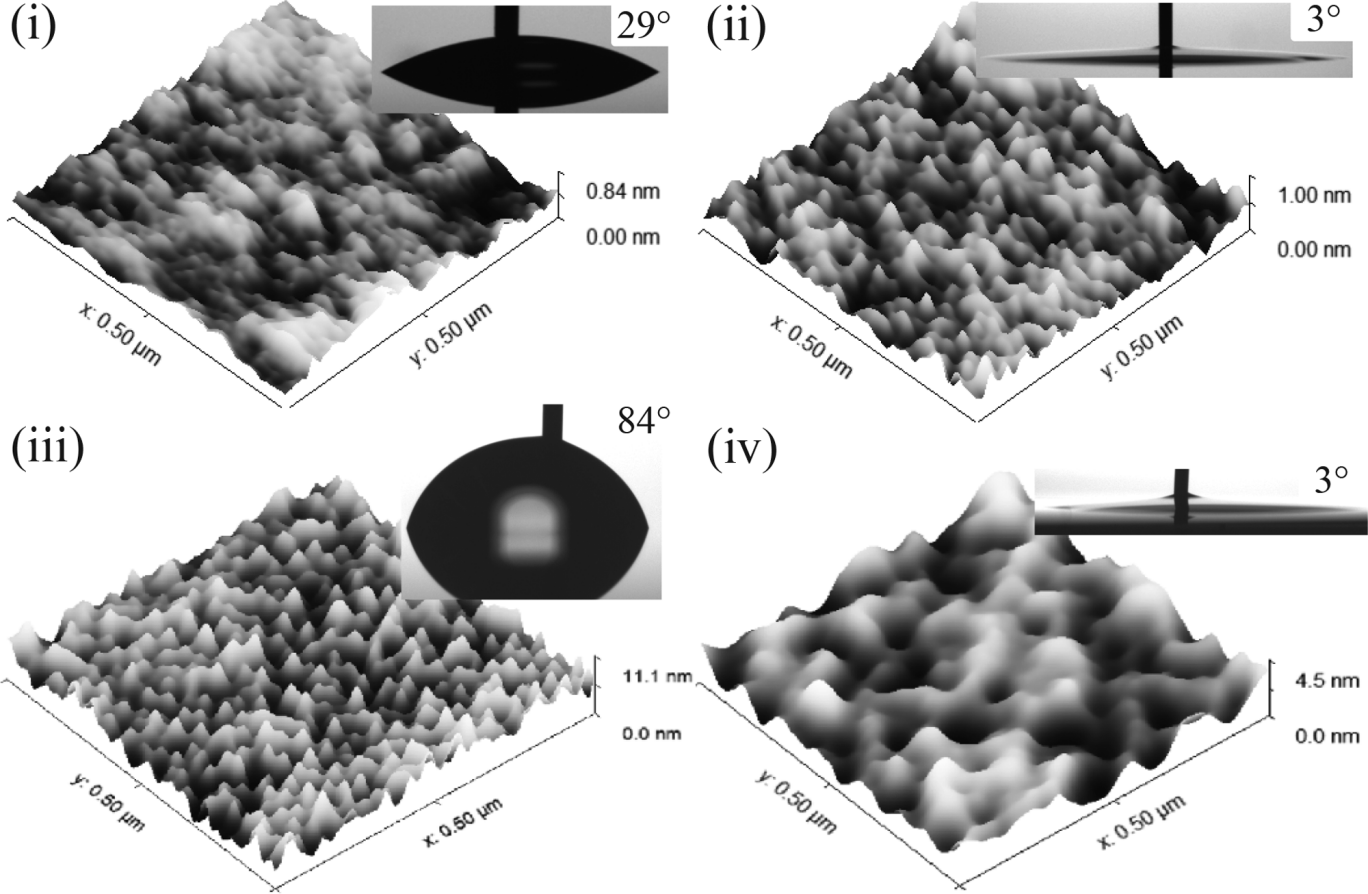
High-resolution AFM scans and water contact angles of (i) an RCA-1 cleaned Si surface after 24 h of exposure to air, (ii) a fresh Ar RIE + RCA-1-activated Si surface, (iii) a pristine AlN thin film surface, and (iv) an SF_6_ + O_2_-activated AlN thin film surface.

In addition, small-area AFM scans of 0.25 μm^2^ were obtained to visually inspect the change in surface morphology at a molecular scale due to the surface activation. The high-resolution AFM scans and their respective water contact angles can be seen in Figure 2.

**Figure 3:**
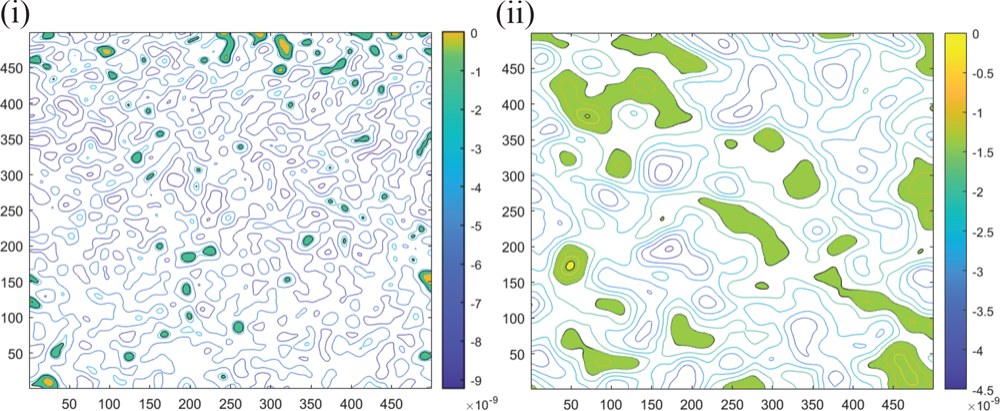
Bearing area computed from the small-area AFM scans shown as solid color contours: (i) nonactivated AlN and (ii) SF_6_ + O_2_-activated AlN.

The total bonding contact area is approximated from the small-area AFM scan—a 0.25 μm^2^ area is analyzed based on the bearing depth of 1.4 nm and the maximum asperity height of 3 standard deviations. Assuming macroscopically flat wavers, on the mirror-polished silicon cap wafer, the bearing ratio is effectively 100%. On the pristine AlN surface, the bearing ratio is 3%, which increases to 15% after SF_6_ + O_2_ activation, as seen in Figure 3i and ii, respectively.

To characterize the chemical species at the surface, XPS measurements were conducted in both survey scan and high-resolution modes. The survey scan reveals an oxygen-rich surface layer on the pristine AlN film, which is altered via plasma activation, as seen in Table 2.

**Table 2:** Effect of Surface Activation on the Chemical Composition of the AlN Surface

	Al (%)	N (%)	O (%)	F (%)	C (%)
pristine	28	18	41	1	12
O_2_	30	21	33	7	9
Ar	30	21	27	13	9
SF_6_	27	21	15	29	8

**Figure 4:**
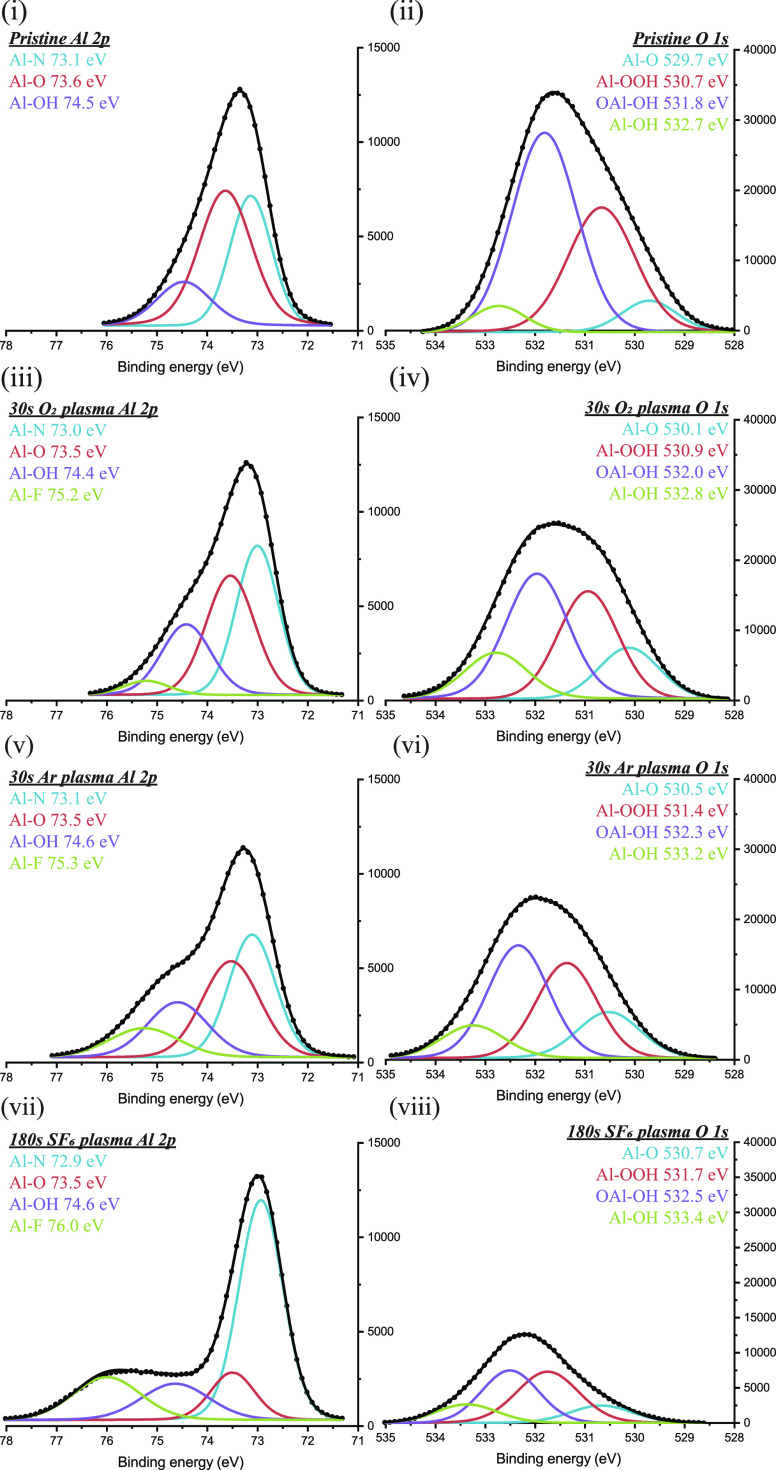
Deconvoluted high-resolution XPS peaks exhibiting the binding energies of both Al 2p and O 1s after various surface treatments: (i and ii) pristine AlN, (iii and iv) 30 second of O_2_ RIE, (v and vi) 30 seconds of Ar RIE, and (vii and viii) 180 seconds of SF_6_ RIE.

The results of the high-resolution scans can be seen in Figure 4. In the high-resolution O 1s peak, excess oxygen content is identified at characteristic energies for hydroxyl binding (Figure 4ii). After either Ar- or O_2_-based RIE activation, the excess oxygen is diminished (Figure 4iv and vi); nevertheless, increased Al–OH binding is identified in the high-resolution Al 2p peak (Figure 4i,iii, and v). Simultaneously, COOH binding decreases in the C 1s high-resolution peak (see the Supporting Information), and a small unidentified subpeak in N 1s binding at approximately 402 eV is removed. Now, the envelope of O 1s (Figure 4iv and vi) is identified as that of aluminum oxyhydroxide. As fluorine is added to the RIE gas mixture, the fluorine concentration increases, whereas the oxygen concentration decreases in an approximate 1:1 ratio (see Table 2). Simultaneously, oxygen-related binding energy is increased throughout the surface region (Figure 4viii), whereas Al–N binding is largely unaffected (Figure 4vii), indicating that fluorine penetrates no deeper than the initial oxygen heavy surface.

**Figure 5:**
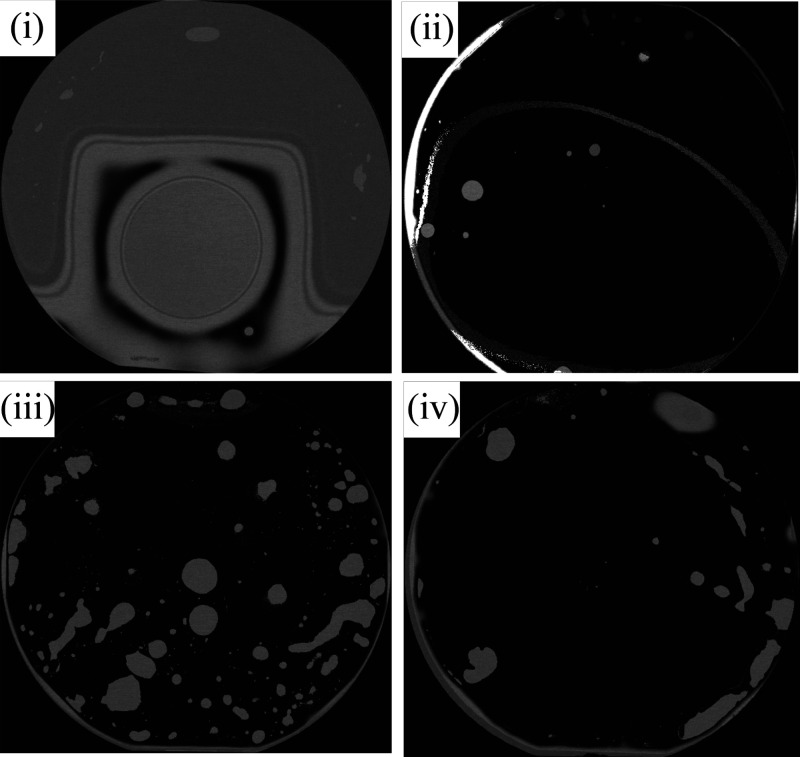
SAM micrographs of the bonds: (i) nonactivated bond (NA), (ii) room temperature bond (RT), (iii) vacuum bond (V), and (iv) RT combined bond (C).

Wafer-level bonding experiments were carried out as summarized in Table 3, with a combination of surface activations and wafer bonding profiles. The four wafer bonding experiments included (i) a nonactivated bond made in the wafer bonder with progressive annealing stages (NA), (ii) a RT bond made in ambient air outside of the wafer bonder (RT), (iii) a vacuum bond made in the wafer bonder with progressive annealing stages (V), and (iv) a combined bond made in the activation process in ambient air but carried out in the wafer bonder with progressive annealing stages (C). The nonactivated bond showed an unsuccessful contact, whereas the remaining three bonds resulted in a range of contact areas observed using SAM. The SAM micrographs in Figure 5 show an unsuccessful contact of nonactivated AlN (i), a successful RT contact of activated AlN (ii), a voided interface in the vacuum contact (iii), and reduced voiding in the combined method (iv).

**Table 3:** Overview of the Wafer-Bonded Samples, Bonding Characteristics, and AlN Properties

	nonactivated bond (NA)	room temperature bond (RT)^a^	vacuum bond (V)	combined bond (C)
activation		SF_6_ + Ar	SF_6_ + Ar	SF_6_ + O_2_ & SF_6_ + Ar
wafer bonding^b^				
*t*_21_	air 1 h	air 48 h	vacuum 1 h	air 5 h
*t*_80_	nitrogen 3 h		vacuum 3 h	vacuum 3 h
*t*_300_	vacuum 4 h		vacuum 4 h	vacuum 4 h
high-temperature (HT) annealing^c^	vacuum 24 h 600 °C	vacuum 24 h 600 °C	vacuum 24 h 600 °C	vacuum 24 h 600 °C
SAM contact^d^/dicing yield (%)	0/10	98/10	86/30	92/90
median tensile strength (MPa)				
pre-HT annealing	3	1	2	14^e^
post-HT annealing		6	9^e^	9
AlN thickness (nm)	910	520	310	720
AlN surface roughness, *R*_q_ (Å)	7	7	6	6
AlN Raman stress (MPa)	600	900	700	600

a Room temperature bond (RT) was made outside of the wafer bonder.

b Wafer bonding atmosphere and time from different periods of the temperature–bonding force–time profile seen in Figure 1ii.

c High-temperature (HT) annealing is the condition of the high-temperature annealing performed in a vacuum furnace.

d SAM contact area is the percent of contact area mapped in the SAM micrographs.

e Samples often exhibited tensile strengths that exceeded the upper limits of the apparatus measuring capability.

**Figure 6:**
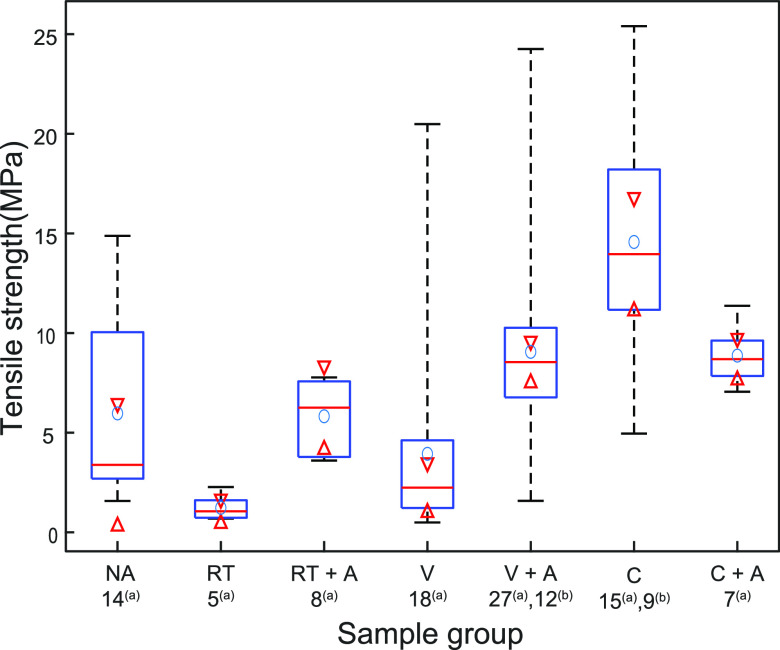
Die-level tensile strength test results, where *n* is the number of independent samples tested for the sample group. NA is nonactivated, *n*_NA_ = 14; RT is the room temperature, *n*_RT_ = 5; RT + A is the room temperature plus annealing, *n*_RT + A_ = 8; V is the vacuum, *n*_V_ = 18; V + A is vacuum plus annealing, *n*_V + A_ = 27; C is combined, *n*_C_ = 15; and C +A is combined plus annealing, *n*_C + A_ = 7. The high-temperature annealing is defined in Table 3. In the figure, superscripts (a) and (b) are the total number of independent samples tested and the number of dies that did not fracture, respectively. The whiskers include 25% of the data on both ends, the blue circle shows the mean, and the red line points at the median, the 95% confidence interval of which is indicated by the red arrows.

The bonded wafers were diced into 25 mm^2^ dies, and the ratio of dies intact was estimated. A summary of the tensile strength results can be seen in Table 3. The SAM yield is based on the percentage of the area in the micrograph that appears black after binary conversion, whereas the dicing yield is based on the number of dies that survived the dicing process. The dies were tensile tested to determine the tensile strength of the interface, and the results can be seen in Figure 6. A subset of dies from a bonded wafer was subsequently annealed in a vacuum at 600 °C to study the impact on the tensile strength.

**Figure 7:**
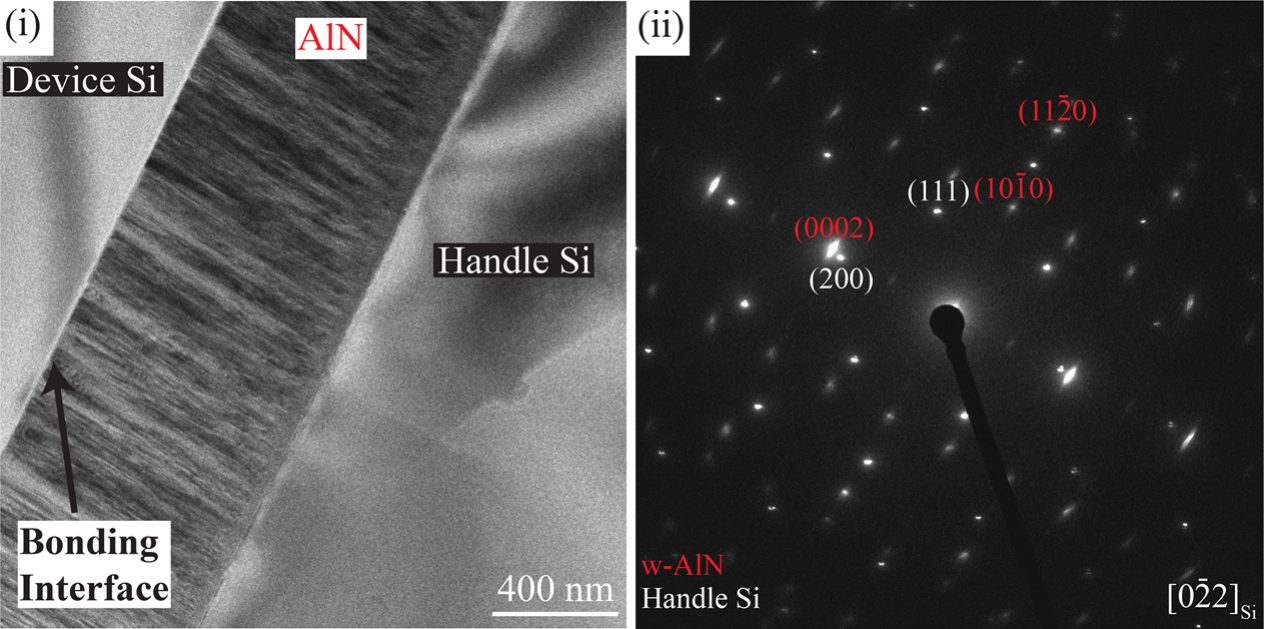
(i) BF-TEM micrograph of the growth and bonding interface and (ii) SAED of the handle Si and AlN layer of the combined bond (C) sample.

As the combined bond resulted in the highest tensile strength, it was chosen to be analyzed using high-resolution TEM and STEM. An overview micrograph of the sample can be seen in Figure 7i, and the corresponding SAED from the handle and sputtered AlN can be seen in Figure 7ii. The SAED pattern showed highly *c*-axis oriented w-AlN, indicating high crystal quality centered somewhat consistently around the handle Si perpendicular direction, Si[100]. In the in-plane direction, AlN does not appear to have any orientation, as both AlN(101̅0) and AlN(112̅0) reflections are visible in the diffraction pattern. This behavior is characteristic of reactive sputtered AlN.

**Figure 8:**
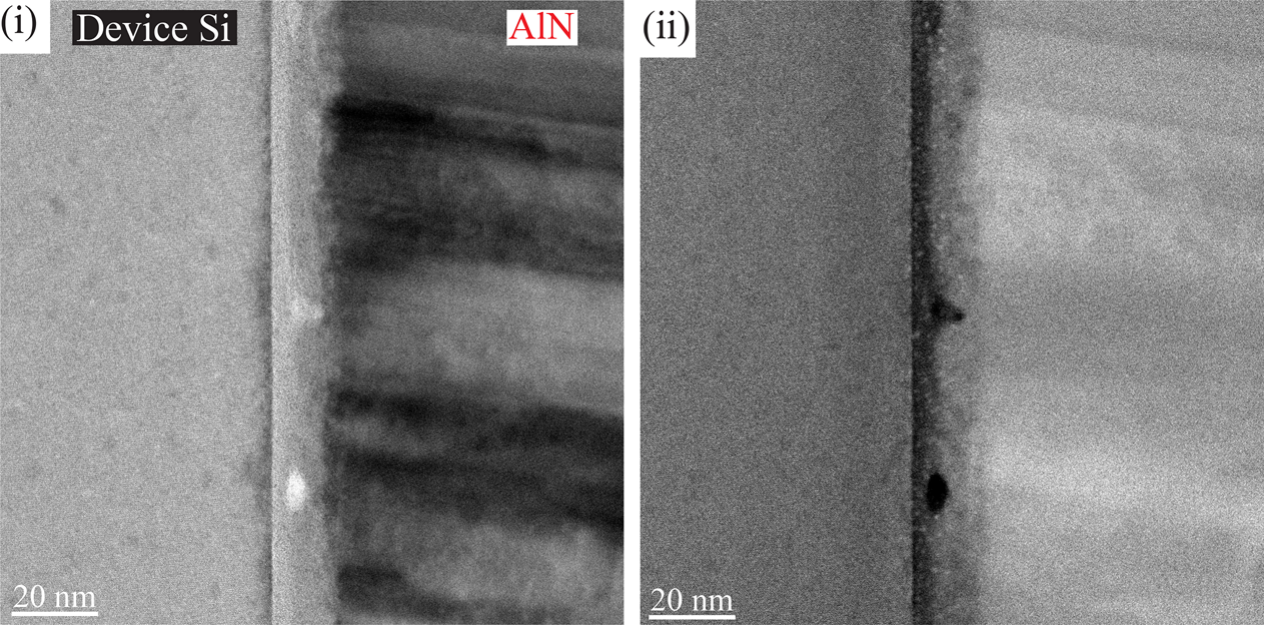
STEM (i) BF and (ii) HAADF micrographs of the Si/AlN bonding interface of the combined bond (C).

Inspection of the bonding interface revealed that there are several layers that form the interface, as seen in Figure 8. This is most noticeably observed in the STEM-high-angle annular dark-field (HAADF) micrograph (Figure 8ii). There is a dark layer with a thickness of 4.84 ± 0.87 nm. The thickness of the layer appears to follow the topography of the mating surface, as near regions of valleys or voids in the layer are thicker. Next to the darker layer, there is a layer of lighter contrast that fades in contrast toward AlN. In addition to the multilayer structure of the bonding interface, several voids were observed along the interface with an approximate void density of 10.6 ± 2.9 voids/μm.

**Figure 9:**
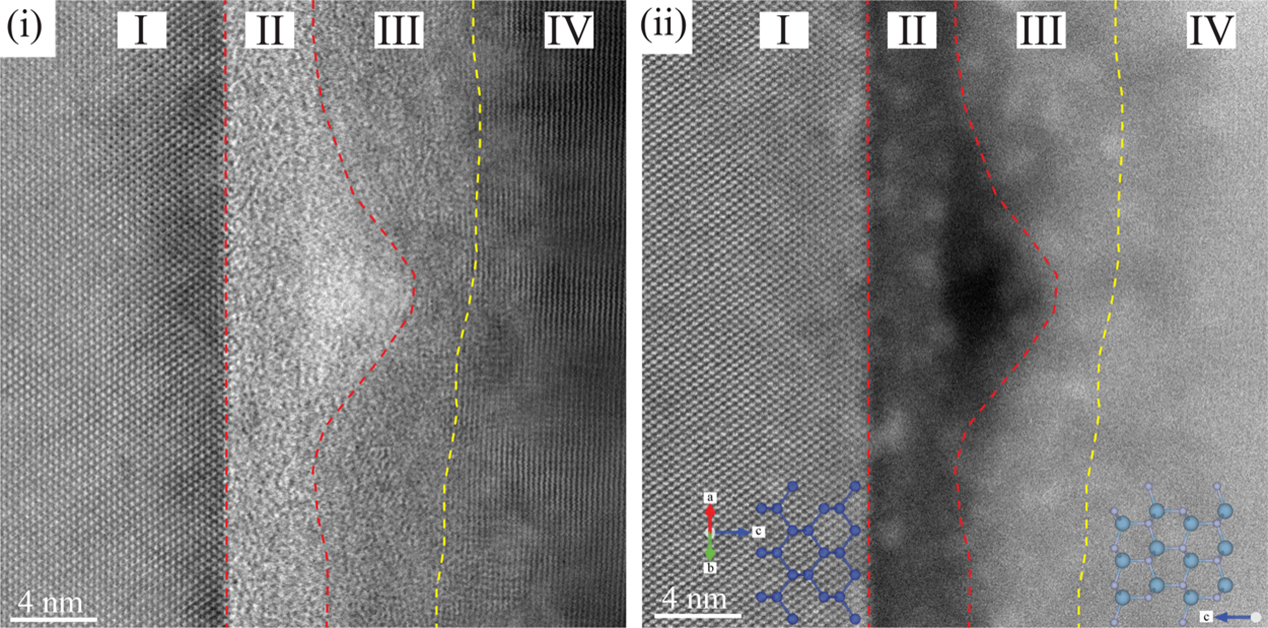
High-magnification STEM (i) BF and (ii) HAADF micrographs of the bonding interface of the combined bond (C). Layers have been shown with layer boundaries indicated with dashed lines.

The interface was inspected at a higher magnification in a region close to a bonding void. The STEM-BF and STEM-HAADF micrographs can be seen in Figure 9. Labeled in the figure are the layers of the bonding interface. The interface between layers III and IV is less well-defined compared to the other interfaces, and hence, the interface has been indicated in the figure with a yellow dashed line. The interface between layers I and II is clearly the Si/SiO_2_ interface, and layer II is entirely amorphous. Layer III is more complex and appears to be a transition layer from the polycrystalline AlN (IV) to the amorphous SiO_2_ layer (II). Small polycrystals are present in an amorphous matrix that constitutes layer III, which finally transitions into fully polycrystalline AlN as it approaches layer IV.

**Figure 10:**
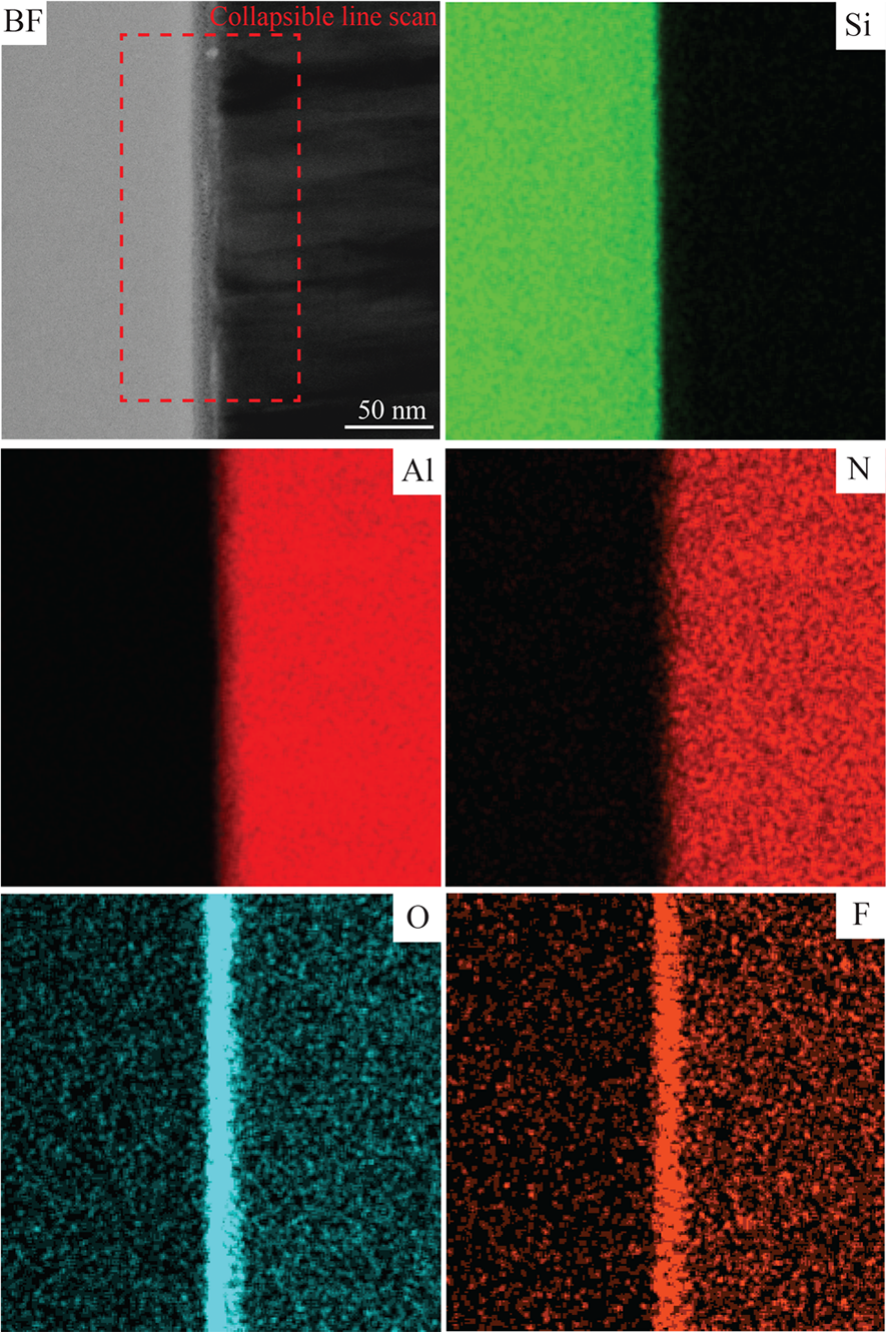
Energy-dispersive spectroscopy (EDS) mapping of the bonding interface of the combined bond (C). Shown in the STEM-BF micrograph is the region of the collapsible line scan in Figure 11.

**Figure 11:**
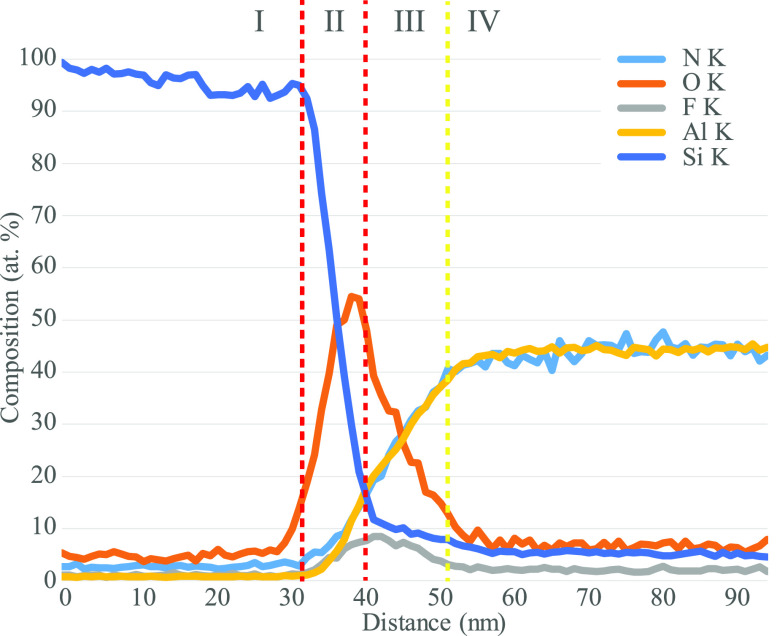
Collapsible EDS line scan from the region indicated in red in the BF-STEM micrograph from Figure 10.

To understand the layered behavior, an EDS analysis was performed. Figure 10 shows an EDS map of the bonding interface, and Figure 11 shows the collapsible line scan generated from the EDS map. The layer boundaries shown in Figure 9 have been superimposed onto the line scan seen in Figure 11. The bonding interface clearly consists of O and F. From both Figures 10 and 11, it is clear that O is present in both layers II and III, whereas F is mostly present in layer III. Therefore, the layer structure and chemical composition of the bonding interface appear to be layer I: Si, layer II: SiO_2_, layer III: fluorine containing ALON, and layer IV: AlN.

## Discussion

In the prebonding characterization stage, the measurements, such as contact angle and surface roughness, showed that superhydrophilic surfaces were obtainable and that the fluorine-based plasma treatment reduced surface roughness in samples that had unacceptably high roughness. Additionally, the AFM micrographs and their related bearing area maps, which were digitally filtered, showed that the surface features are smoother and that the bondable regions coalesce. The advantages of enhanced surface features from the fluorine-based plasma treatment, from a process point of view, are integration of surface activation and prepolishing, which are required for hydrophilic direct bonding processes. Despite these advantages, fluorine contamination in microelectronics is a well-known issue^24,25^ and could not only possibly result in the degradation of devices fabricated on the alternative SOI platform but also potentially degrade the bonding integrity. Nonetheless, with further investigation of fluorine-based plasma treatments and the long-term reliability, it is found that the potential exists for the elimination of costly prebonding chemical–mechanical polishing of bonding surfaces.

XPS data of the surface activation processes showed that fluorine replaces oxygen at the surface region, nitrogen remains largely unaffected, and the concentration of carboxylic acids is reduced. Since solvent cleaning is not sufficient to remove all of the tightly bound carbon, such as carboxylic acids, more aggressive cleaning protocols are utilized, such as RCA-1 on the silicon surface. However, the aggressive wet cleaning protocols tend to attack the grain boundaries of AlN, resulting in very high surface roughness. Thus, argon and oxygen plasmas are a better means of removing the contamination. However, in this study, neither argon nor oxygen was sufficient to both smoothen the surface topography and induce a hydrophilic surface. Hence, fluorine was added to the activation gas in relatively large quantities to form a layer of aluminum oxyfluoride that was capable of both increasing the bearing area and forming the intersurface bonds.

It is clear from the bonding results that hydrophilic wafer bonding is multiparameter and complex in nature. Bonding results, such as the SAM micrographs and the variation in tensile strength measurements, show large variations between samples that cannot be attributed to the bonding process alone. Large voids observed in the SAM micrographs are the result of a combination of several factors, including surface contamination, surface topography, and trapped H_2_ from the polymerization reaction, to name a few. In the case of the vacuum bonded sample, voids formed at the bonding interface could be due to the plasma interaction with the surface and/or cleaning process undertaken preceding the surface activation and subsequent outgassing. The direct bonding process is very sensitive to contamination and topography, where these effects can result in voids tens to hundreds of microns in size. The processes in this work are not optimized for defect-free interfaces; however, every effort has been made to reduce the impact of macrosized voids on the analysis.

The mechanical assessment of the bonded samples revealed a clear difference between the nonactivated and activated surfaces. It should be highlighted that the SAM yield and the dicing yield are not correlated and that the contrast difference in the SAM micrographs is from both noncontacted and contacted regions. In the SAM micrograph of the nonactivated sample, there is a clear indication that there was not a properly wetted contact interface, due to the hydrophobic nature of the AlN surface. The lack of wetting and bonding was also confirmed by a very low dicing yield. The other bonded sample that experienced a relatively low dicing yield was the surface-activated sample bonded in room temperature ambient air, indicating that the polymerization was not sufficient, although strong hydrogen bonding mediated by water molecules was achieved. Moreover, the tensile strength was improved after high-temperature annealing, most likely due to the high temperature allowing interfacial reactions to proceed.

Tensile strength and fracture surface analysis included the qualitative analysis of the fracture interface using a light microscope, where a significant number of bonded samples were tested. Not all of the dies fractured during the tensile strength tests as the physical limit of the measurement equipment varies between 10 and 20 MPa, which has previously been reported as the tensile strength of more traditional Si–SiO_2_ direct bonds from the literature^26−28^ and verified using the same tensile measurement setup.^6^ Due to the upper limit of the test setup, the maximum tensile strength of some test groups remains undetermined, which was observed, for example, with the combined (C) sample group. Nonetheless, this result indicates that AlN–Si bonding presented in this work matches or exceeds tensile strengths reported for the Si–SiO_2_ directly bonded counterpart, even when factoring in the voids detected at the interface.

## Conclusions

In conclusion, this work has presented an alternative SOI platform built on a reactively sputtered AlN layer. This work examined the impacts of different plasma treatments on the surface properties of AlN, such as hydrophilicity, chemical state, and topography. Additionally, hydrophilic direct wafer bonding was carried out on a range of AlN-surface-activated films. The interfacial bonding quality was assessed using SAM, and the tensile strength of bonded samples was measured. Finally, the sample that exhibited the highest tensile strength was characterized using high-magnification microscopy.

The results of the work showed that it was possible to surface activate AlN to form a hydrophilic surface, a requirement of hydrophilic direct bonding. Additionally, treating AlN with fluorine-based plasma treatments effectively improved not only the hydrophilicity but also the surface roughness and nanotopography, enabling a more bondable surface. From the XPS and STEM results, it appears that the fluorine species together with the hydroxides in the surface region initiates the hydrophilic bonding to the hydroxides on the opposing Si surface. Tensile strength measurements of wafer-bonded samples revealed a range of results, which depended on the surface activation. The most promising sample demonstrated tensile strength values comparable to traditional SiO_2_-based SOI.

## Supporting Information Available

Results of XRD 2D 2θ-χ diffraction maps from a characteristic AlN film; Raman spectrum after the AlN deposition; linearized Raman stress in the group of AlN films as determined from the *E*_2_^2^ phonon mode; additionally, sputtered AlN topography using a spectroscopic ellipsometer; AFM depiction of the surface waviness; and high-resolution XPS spectra of both carbon and nitrogen for selected AlN surface plasma treatments (PDF)
