# Supplementary material for: Aluminum Nitride to Silicon Direct Bonding for an Alternative Silicon-On-Insulator Platform
Source: ACS Appl Mater Interfaces. 2021 Aug 4;13(32):38857–65. doi: 10.1021/acsami.1c09535 (PMC8397240; doi:10.1021/acsami.1c09535)
Supplement: Supplementary file 1 — am1c09535_si_001.pdf [file am1c09535_si_001.pdf]

Supporting Information

Aluminum Nitride to Silicon Direct Bonding for an Alternative Silicon-On-Insulator Platform

Jani Kaaos, Glenn Ross*, and Mervi Paulasto-Kröckel

Department of Electrical Engineering and Automation, Aalto University, P.O. Box 13500, FIN-00076 Aalto, Finland.

*Corresponding author: Glenn Ross E-mail address: glenn.ross@aalto.fi.

Figure S1 shows an example of a 2D 2θ-χ diffraction map from the group of AlN films. The film grows along c-axis parallel to the Si (100) face of the substrate, although, there is a slight tilt angle is noticeable. Figure S2 shows the change in the Raman peaks after the AlN deposition; herein, the bias in the ω-axis is not corrected for. Figure S3 shows linearized Raman stress in the group of AlN films as determined from the E_2^2 phonon mode utilizing common literature values[1]. Figure S4 shows a common case of AlN thin film topography in the group of films as characterized via SE. Figure S5 shows an example of the waviness component in a large area AFM scan. Figure S6 shows the effect of the rudimentary plasma treatments as characterized by XPS on both carbon and nitrogen binding.

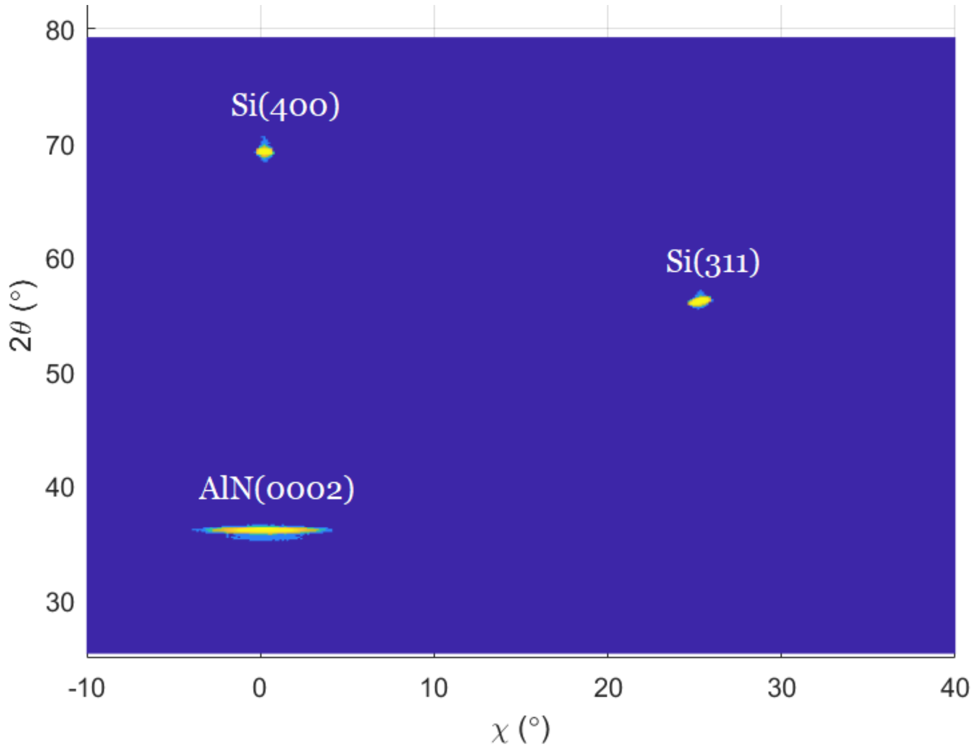

Figure S1: 2D 2θ-χ diffraction map of the scanned XRD angles

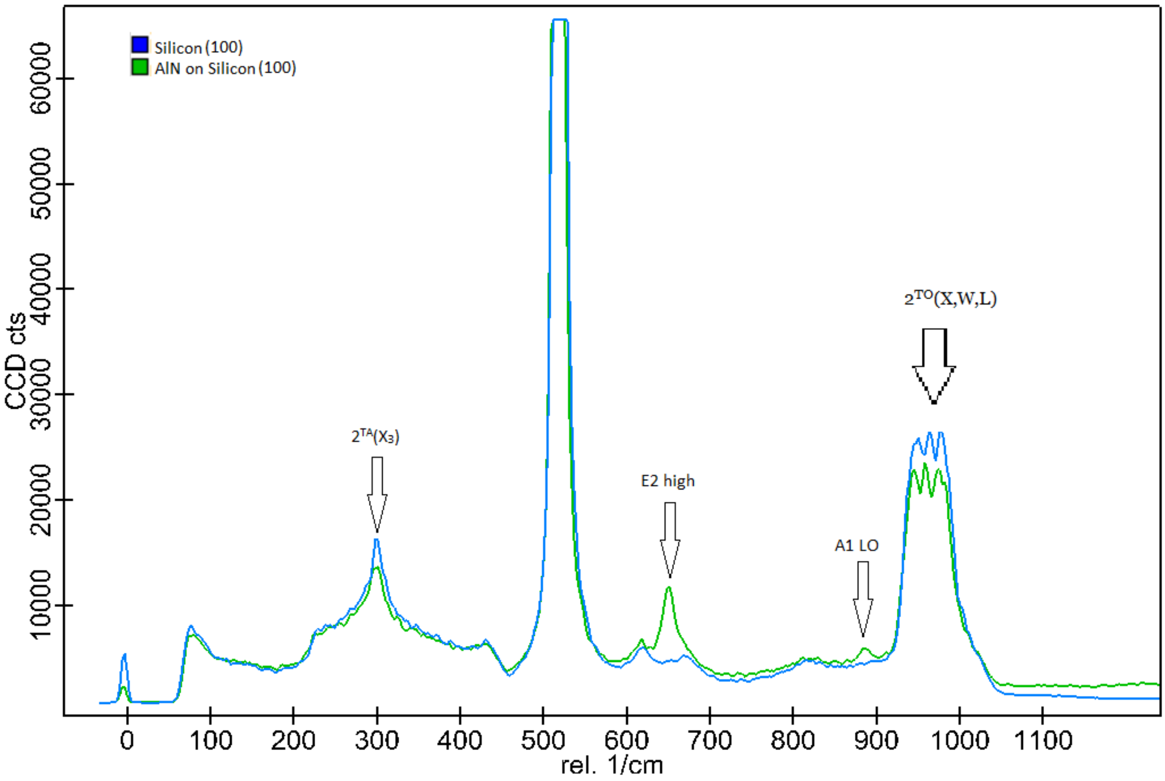

Figure S2: Raman spectrum of a Si/AlN stack superimposed onto the Raman spectrum of the pristine Si substrate

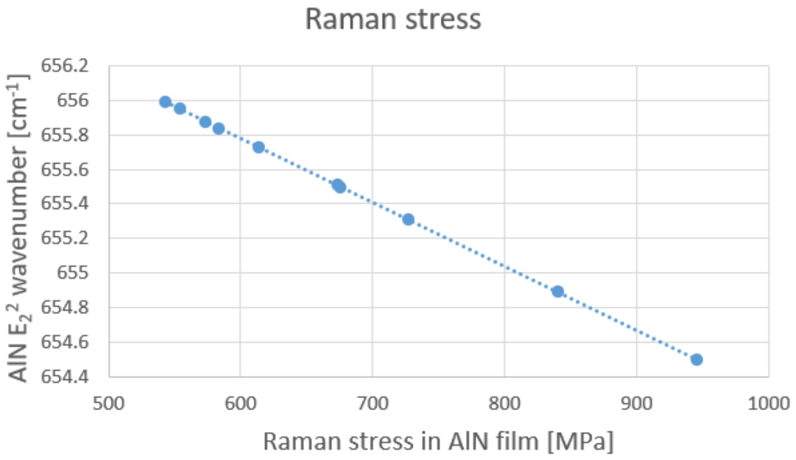

Figure S3: Raman stress approximated from the E_2^2 phonon mode utilizing fitting parameters ω_0 = 658 cm^{-1}, C_E = 3.7 GPacm

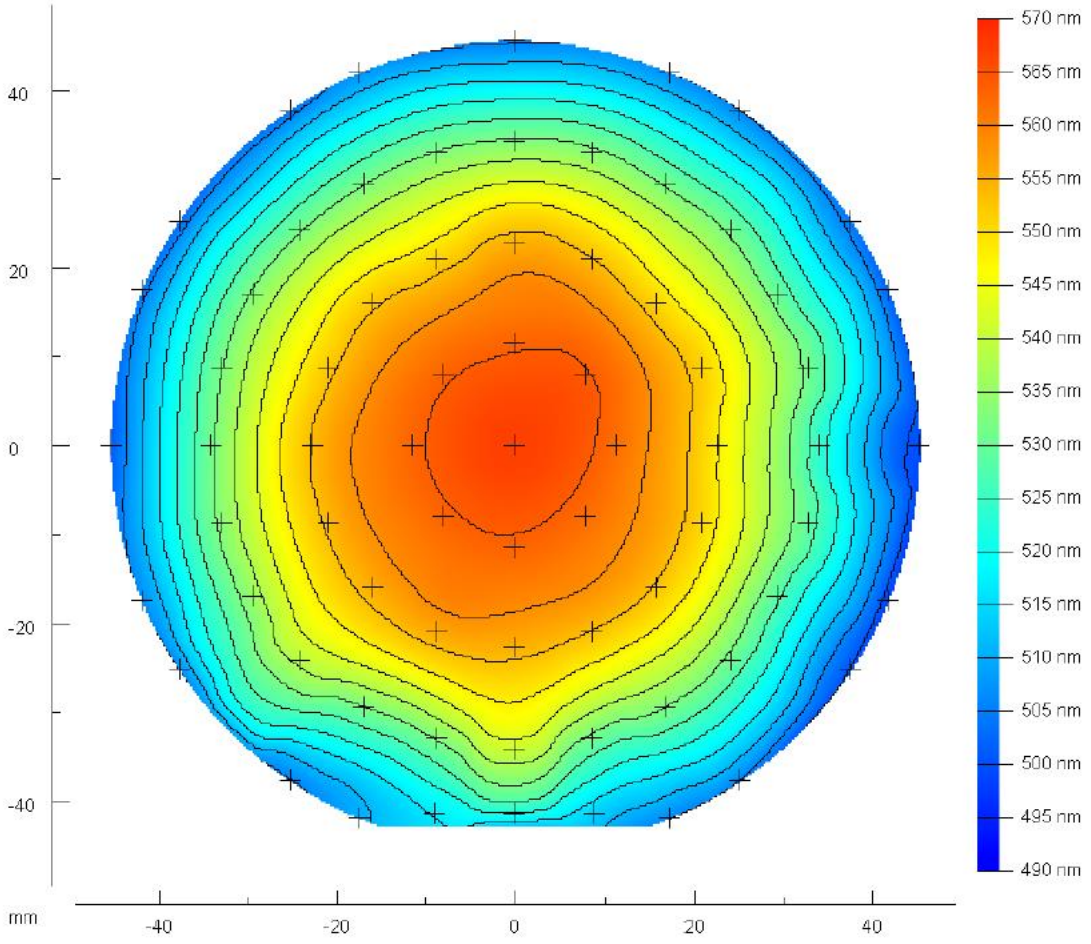

Figure S4: A depiction of the topography of the sputtered AlN films

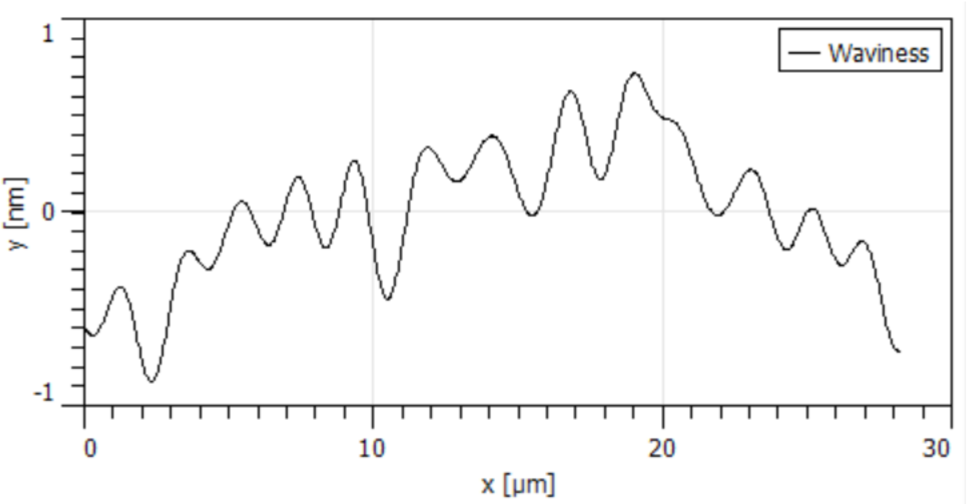

Figure S5: A depiction of the surface waviness component of the sputtered AlN films

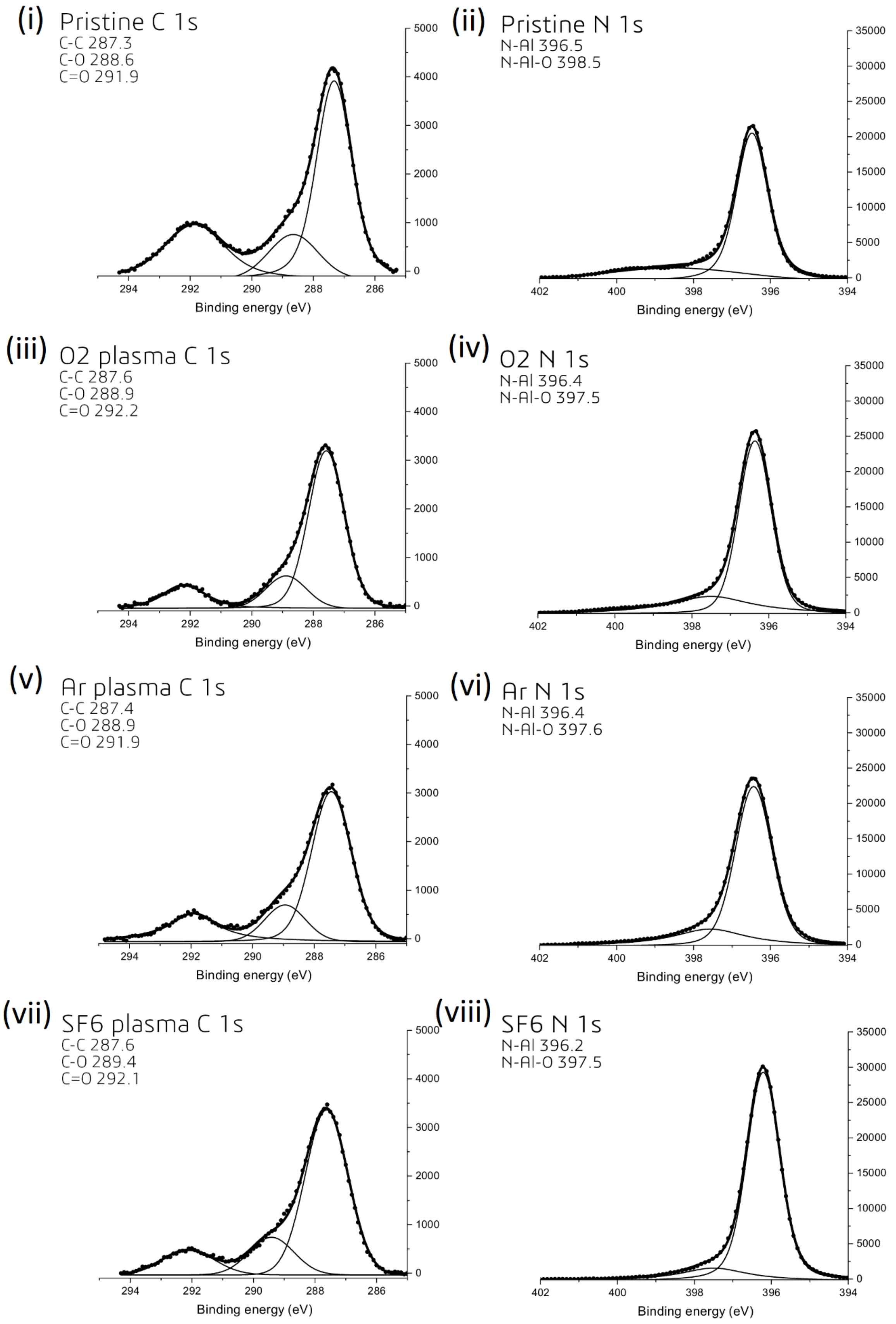
(i)
Pristine C 1s
C-C 287.3
C-O 288.6
C=O 291.9
(ii)
Pristine N 1s
N-Al 396.5
N-Al-O 398.5
(iii)
O2 plasma C 1s
C-C 287.6
C-O 288.9
C=O 292.2
(iv)
O2 N 1s
N-Al 396.4
N-Al-O 397.5
(v)
Ar plasma C 1s
C-C 287.4
C-O 288.9
C=O 291.9
(vi)
Ar N 1s
N-Al 396.4
N-Al-O 397.6
(vii)
SF6 plasma C 1s
C-C 287.6
C-O 289.4
C=O 292.1
(viii)
SF6 N 1s
N-Al 396.2
N-Al-O 397.5
Binding energy (eV)

Figure S6: High-resolution XPS spectrum of both carbon and nitrogen for (i,ii) pristine AlN film, (iii,iv) oxygen plasma treated AlN film, (v,vi) argon plasma treated AlN film, (vii, viii) SF_6 plasma treated AlN film.
